# Spatial prediction of dog population distribution in Kenya

**DOI:** 10.1371/journal.pone.0343347

**Published:** 2026-04-13

**Authors:** Moumita Das, Maria Sol Perez Aguirreburualde, Shepelo Getrude Peter, Anima Sirma, Andres Perez

**Affiliations:** 1 Center for Animal Health and Food Safety, College of Veterinary Medicine, University of Minnesota, Saint Paul, Minnesota, United States of America; 2 Department of Veterinary Population Medicine, University of Minnesota, Saint Paul, Minnesota, United States of America; 3 Kenya Women Veterinary Association and Department of Clinical Studies, Faculty of Veterinary Medicine, University of Nairobi, Nairobi, Kenya; 4 Directorate of Veterinary Services, Kenya Ministry of Agriculture and Livestock Development, Nairobi, Kenya; Xinjiang Medical University Affiliated First Hospital, CHINA

## Abstract

Free-roaming dogs pose a significant public health concern due to their role in disease transmission. Rabies has been endemic in Kenya for over a century, yet a sustainable and standardized method for estimating dog populations remains unestablished. To address this gap, we applied kriging and co-kriging spatial interpolation techniques to predict the distribution of free-roaming domestic dogs across different Kenyan counties. To improve the model’s accuracy, we incorporated environmental and demographic predictors such as daily temperature, the Normalized Difference Vegetation Index (NDVI), and human density. Dog population data at the village level were collected from 34 counties through an online survey of veterinary professionals in both the public and private sectors. A spherical model was used to construct the semivariogram, integrating temperature, NDVI, and human density to refine spatial predictions. Kenya’s total free-roaming dog population was estimated to be 7.46 million. The density of dogs per square kilometer varied across counties, corresponding to a median national density of 12.13 dogs per square kilometer. Compared to models incorporating no or only single or multiple covariates, the co-kriging model incorporating human density provided the best fit, with the minimum estimated difference between observed and predicted values. The spatial distribution map highlights arid and sparsely populated pastoral counties having lower dog densities, whereas peri-urban, densely populated, and agricultural counties exhibit higher dog numbers. This study provides a spatial framework for estimating free-roaming dog populations, which can inform the design and implementation of rabies control programs and public health interventions in Kenya and other infected countries.

## Introduction

Domestic dogs are among the most well-known companion animals, forming a mutualistic relationship with humans. Although they are primarily domesticated and rely on human care, when allowed to roam freely, they display behaviors such as extensive roaming, scavenging for food, and defending territories [1]. Their unrestricted movement and frequent interaction with humans, other domestic animals, and wildlife, make dogs a relevant reservoir for disease transmission. According to the World Health Organization (WHO), free-roaming dogs in many low- and middle-income countries are responsible for 99% of human rabies cases, primarily through bites and scratches [2].

Rabies is a virus-borne zoonotic disease that affects a wide range of mammalian hosts. The virus exists in two distinct patterns, referred to as urban and sylvatic cycles [3]. Urban rabies primarily circulates within the street and among community dogs, which then transmit the virus to free-roaming owned and pet dogs, posing a serious risk to public health. In turn, the sylvatic cycle is prevalent in wildlife. Asia accounts for the highest proportion of rabies-related human deaths, at 59.6%, followed by Africa at 36.4% [4]. The socioeconomic burden of rabies is particularly high in these regions. In Africa, around 21,000–25,000 human fatalities occur because of dog-mediated rabies [5]. Globally, the estimated disability-adjusted life years (DALY) due to rabies is 8.6 billion USD (95% CIs: 2.9–21.5 billion), representing the total years lost due to premature death and the loss of workforce productivity caused by poor health [2].

Given these challenges, understanding the size and distribution of the dog population is crucial for designing effective management and disease control programs and, most importantly, the design of vaccination campaigns. Free-breeding dogs, often owned but allowed to roam pose a significant rabies risk in developing countries and are estimated to number around 500 million, representing nearly half of the global dog population. [6]. Estimating dogs’ numbers and spatial distribution is common, with various studies employing different spatial methods. For instance, research conducted in Brazil and Chad applied kernel density methods to map the spatial distribution of stray dogs [7,8]. Other studies have utilized approaches such as a Bayesian N-mixture model in the UK [9] and a Bayesian spatial regression model in Thailand [10]. Some other standard techniques are available for dog population enumeration, such as in Tanzania, where the size of the dog population was predicted to be 2.32 million by utilizing transect data [11]. Thailand estimated 12.8 million dogs through a random forest model [12]. Besides, some other simple methods like the photographic sight-resight method in Afghanistan and Indonesia [13,14], the Chapman mark-resight method in India [15], and a remote sensing technique using Google Street View in Peru [16] have been employed in counting the free-roaming dogs. In the current study, we utilize a kriging/co-kriging approach to estimate dog population numbers, leveraging its ability to interpolate spatial data while incorporating environmental covariates [17]. Unlike previous methods, which rely on direct counting or statistical modeling without accounting for spatial autocorrelation, kriging/co-kriging offers a geostatistical framework that enhances prediction accuracy by utilizing spatial dependence. While random forest and other models are powerful for modeling complex, non-linear relationships between predictors and population density, they do not inherently account for spatial autocorrelation, the principle that locations closer to one another are more related than those farther apart. Given that the data in this study consists of discrete survey points, the primary objective was to interpolate values for all unsampled locations across Kenya to create a continuous prediction surface. Geostatistical methods like kriging and co-kriging are specifically designed for this purpose, as their predictions are explicitly based on the spatial dependence structure of the data, which is captured in the semivariogram. Co-kriging was therefore selected as the most appropriate method for this spatial interpolation goal.

In Kenya, however, definitive national data on dog populations remain scarce. Although a 2017 report by the Zoonotic Disease Unit estimated the population at 5–6 million [18], most available information comes from localized studies. For instance, a study in Machakos county reported dog densities ranging from 6 to 110 dogs per square kilometer, depending on whether the area is rural or urban [19]. Another study in western Kenya estimated 54 dogs per square kilometer with a human-dog ratio of 7:1 [20]. In Laikipia county, the size was estimated at 34,275 by calculating the average number of dogs per household [21]. To address this gap, our study leverages geostatistical interpolation techniques, specifically kriging and co-kriging, to estimate the spatial distribution of dog populations across Kenya. While co-kriging is rarely used in free-roaming dog population studies, its successful application in modeling human density [22], assessing invasive aquatic species risks [23], estimating COVID-19 cases [24], and predicting foot and mouth disease outbreaks [17] suggests its potential value in this study.

The objective of this study was (1) to estimate the spatial distribution of dog populations in Kenya using geostatistical interpolation techniques, specifically kriging and co-kriging and (2) to incorporate environmental covariates (e.g., higher daily temperature, NDVI, and human density) in the co-kriging model to improve prediction accuracy and assess their influence on dog population distribution. To our knowledge, this is the first study to systematically estimate dog populations at the national level in Kenya using a geostatistical interpolation technique. The information presented here will support the design and implementation of disease control measures, most notably vaccination campaigns, in the country and could serve as a model for other countries in the region.

## Methods

### The administrative levels of Kenya

Kenya, located in East Africa, shares borders with Uganda, South Sudan, Ethiopia, Somalia, and Tanzania. The country’s administrative structure consists of one national and 47 county governments. Each county is further subdivided into sub-counties, wards, and villages. Approximately 62% of Kenya’s land area is occupied by pastoralist communities, which comprise only 12% of the country’s population. The most densely populated counties include Mombasa, Nairobi, Kisumu, Machakos, Kiambu, Uasin Gishu, Nakuru, and Kajiado. Around 31% of Kenya’s population (around 14.8 million people) resides in these urban areas.

### Data collection

In Kenya, there is no publicly available online data on the population of free-roaming domestic and street dogs. Four webinars titled “Effective Rabies Control Strategy in Domestic Dogs: A Collaborative Approach” were held every Monday of August 2024, as part of our capacity-building efforts in the region and collaboration with the Kenyan Veterinary Board (KVB) and the Kenya Women Veterinary Association (KWVA). The call for the webinars was open to all registered public and private veterinarians and para-veterinarians in Kenya. The first session served as an introductory event, emphasizing the importance of rabies control in domestic dog populations. The subsequent three webinars featured expert discussions led by rabies and public health specialists from the USA, Switzerland, and Kenya. To ensure active participation, only attendees from the first session who completed the survey were eligible to attend the remaining three webinars and earn Continuous Professional Development (CPD) credits. Participants were awarded one CPD credit per hour, with each of the three expert-led webinars lasting two hours, allowing a maximum of six CPD credits. As part of the capacity-building and educational training activity, an anonymous questionnaire was administered to collect data on the participant’s service location, geographical coordinates, estimated number of dogs in their service location, and seasonal variations in dog populations across four times of the year (January-March, April-June, July-September, and October-December). Because precise dog population figures were unavailable, participants provided estimates based on their professional judgment and experience. In cases where they were uncertain, they were encouraged to approximate the number of dogs by multiplying the estimated number of households in the village by the assumed number of dogs per household. Data were organized in a table depicting the geographical location and number of dogs, which helped discussion activities in the capacity-building program and subsequently served as the primary data source analyzed in this study.

All the data involved in this study were collected as part of a training activity. Because data were gathered in the context of an educational activity, the activity was not considered research by the University of Minnesota Institutional Review Board (IRB) Committee, and the follow-up questions do not apply.

### Data transformation and covariates

Dog counts collected through the questionnaires were converted into density values. As the density counts were not normally distributed, we performed a log transformation of this outcome data. However, because village-level land area data for Kenya is not readily available online, we approximated the area of each village using publicly available online measurement tools based on the provided geo-coordinates.. These measurements were then documented in square kilometers for further analysis. It is important to note that this process was only for calculating numerical area values; no copyrighted basemaps (e.g., Google or Esri imagery) were used in the generation of the final figures. The Kenyan boundary shapefile and additional predictor variables were sourced from online databases (Table 1). In the predictor list, we considered higher daily temperature [25], Normalized Difference Vegetation Index (NDVI) [26], and human population density [27]. These factors were selected based on their potential ecological and socio-environmental influences on dog populations. Daily high temperatures can affect dog abundance by promoting increased breeding activity, higher visibility and movement, enhanced food availability, improved survival rates, and more frequent human outdoor interactions, which may facilitate roaming behavior [25]. NDVI is widely recognized for its ability to predict species distribution, abundance, and ecological patterns over space and time, making it a relevant factor in assessing dog presence [26]. Human population density is a strong determinant of domestic dog populations, as dogs are often closely associated with human settlements [27].

**Table 1:** Sources of predictors used in the model, including corresponding web references.

Predictors	Source of data	Reference
Administrative level of Kenya	HDX – Kenya Subnational administrative boundaries	https://data.humdata.org/search?q=kenya+map&ext_search_source=main-nav
Historical climate data -bioclimatic variables (30 seconds)	WorldClim version 2.1	https://www.worldclim.org/data/worldclim21.html #
Normalized difference vegetation index (NDVI) – Raster 300 m	Copernicus land monitoring service	https://land.copernicus.eu/en/products/vegetation/normalized-difference-vegetation-index-300m-v1.0
Population density	EnergyData.Info	https://energydata.info/dataset/kenya-population-and-household-dataset

Kenya’s subnational administrative boundary data includes county-level borders, which are updated annually. Topographic errors were corrected using the ArcMap Integrate tool, with a 30-meter tolerance applied to maintain a high level of spatial resolution. Bioclimatic variables are calculated from monthly temperature and rainfall data at a high-resolution of around 1 square kilometer in a 5-minute grid scale [28]. These variables capture long-term climatic patterns, including monthly minimum, maximum, and average temperatures, precipitation, solar radiation, vapor pressure, and wind speed. The data is aggregated over 1970–2000, comprehensively representing climatic conditions across this timeframe. The current study only included the maximum temperature of the warmest month (BIO5) by considering Kenya’s varied topography, ranging from arid and semi-arid lands (ASALs) in the north and east to highland and lake basin ecosystems. Temperature extremes significantly influence dog distribution across different ecological zones in Kenya.

### Data analysis

To achieve our study goal, a co-kriging model was used to predict the total number of dogs in Kenya. Kriging is a geostatistical interpolation technique widely used for non-random and imperfect data [17]. We developed eight candidate models: one using ordinary kriging without covariates, three universal kriging models, each incorporating a single covariate (high daily temperature, NDVI, or population density), three models combining two covariates (high daily temperature with NDVI, high daily temperature with population density, and NDVI with population density), and one universal kriging model that included all covariates together. To identify the most suitable covariate for co-kriging, model performances were evaluated using the D-value, which quantifies the difference between observed and predicted values for each model. Human density was retained as the sole covariate in the final model due to its superior predictive accuracy, as evidenced by the lowest D-value among all candidate models. Ordinary kriging is used with the assumption of constant mean and variance of the values across the spatial field.


Z(s) = μ + ϵ(s)

Where Z(s) represents the observed values at the location “s,” “μ” is an unknown constant mean, and ε(s) is the spatially correlated residual. On the other hand, the universal kriging is not restricted to the assumption of stationarity, allowing a spatial trend in the mean values μ(s) [29,30].


Z(s) = μ(s) + ϵ(s)

We generated an empirical semivariogram to illustrate how data values change with increasing distance between locations, offering a visual representation of spatial dependence within the dataset [31]. In the kriging model, we used a spherical variogram, a common and robust model for environmental data, representing a gradual increase in spatial variability up to the range, for all eight candidate models. The range represents a solid point at a certain distance until when the data are spatially correlated, indicating that beyond this distance, the data points are not significantly related to each other [32]. We used a semivariogram for ordinary kriging and covariance for other co-kriging models in the model function. The model considered a minimum neighbor of two and a maximum neighbor of five with the one-sector algorithm. One sector considers all the nearby candidate points within a search and threatens them equally without any directional bias.

### Goodness-of-fit for the candidate models

Goodness-of-fit for each candidate model was performed by comparing the observed and predicted values in all 86 locations of dog presence at the village level. For each candidate model, the difference (D-value) between the observed and predicted values was calculated in each tercile. After that, we compared the output of all the 3x2 contingency tables. The lowest D-value produced by a particular model was considered the best-fit model for predicting the dog population in Kenya. The lowest D-value indicates how close the predicted values are to the observed values. We also checked if the D-value of the best-fit model differed more than 10% from the next lower D-value of the candidate model.

### Estimation of dog counts

After recognizing the best-fit model, a summary statistics table was generated in ArcGIS Pro, including count, area, mean, and standard deviation for all 47 counties in Kenya. The count symbolizes the number of raster grids within each county, while the area indicates the total land area in square meters. The mean value reflects the log-transformed dog density per square kilometer. We applied an exponential transformation to the log values to derive the actual dog density. County-level dog populations were then estimated by multiplying the exponentiated dog density per square kilometer by the total county area and dividing by 1,000,000. Finally, the total dog population in Kenya was obtained by summing the estimated dog counts across all counties.

All the maps were generated using the ArcGIS Pro (Version 3.1.0) (ESRI Inc., Redlands, CA, USA) software.

## Results

### Study participants

**Fig 1:**
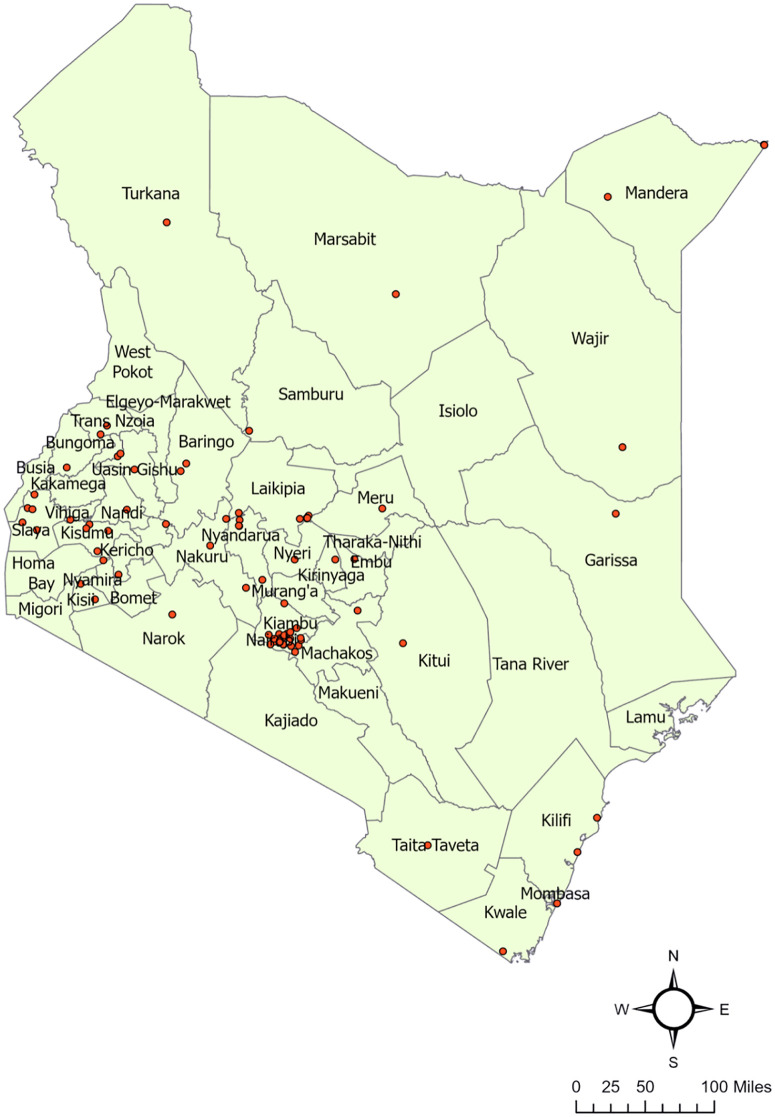
Map of Kenya depicting locations from where data on dog population were obtained (red dots).

Initially, we gathered 109 responses through a questionnaire survey. However, after eliminating outliers and duplicate entries, the final dataset comprised 86 observations. Among these participants, 33 (38.4%) were public veterinarians, 11 (12.8%) were private veterinarians, 15 (17.4%) were para-veterinarians, and 6 (7.0%) were categorized as others. Others included three veterinarians associated with academia, one veterinary laboratory technician, one veterinarian in a pharmaceutical company, and one individual whose affiliation was not specified. The data covered 34 out of 47 counties, with multiple responses collected from some counties (Fig 1**).**

### The best-fitted model

When comparing ordinary kriging and universal co-kriging models, the model incorporating human density only as a covariate best fitted the observed data, as indicated by the lowest D-value (0.94) estimated. The next closest D-value was obtained from the model with NDVI (D-value = 1.39), which was 47.87% higher, followed by the model with temperature (D-value = 1.46), which was 55.32% higher, both exceeding 10% difference. Based on these results, we selected the best-fitting model, which considered human density as the only covariate (Table 2). The 33rd percentile (Q1 threshold) for this model was 3.08, while the 66th percentile (Q2 threshold) was 3.97. The risk terciles (Q1, Q2, and Q3) were categorized based on these thresholds.

**Table 2:** The difference between the observed and expected number of dogs (D-value) in different kriging and co-kriging models.

Type of Models	D-value
Human density + Temperature + NDVI	1.83
Temperature	1.46
NDVI	1.39
Temperature + NDVI	2.45
Human density	0.94*
Human density + Temperature	3.06
Human density + NDVI	3.29
Ordinary	1.87

* The lowest D-value (difference between the observed and expected number of dogs) indicating the best fitted model

### Estimating total dogs in Kenya

**Fig 2:**
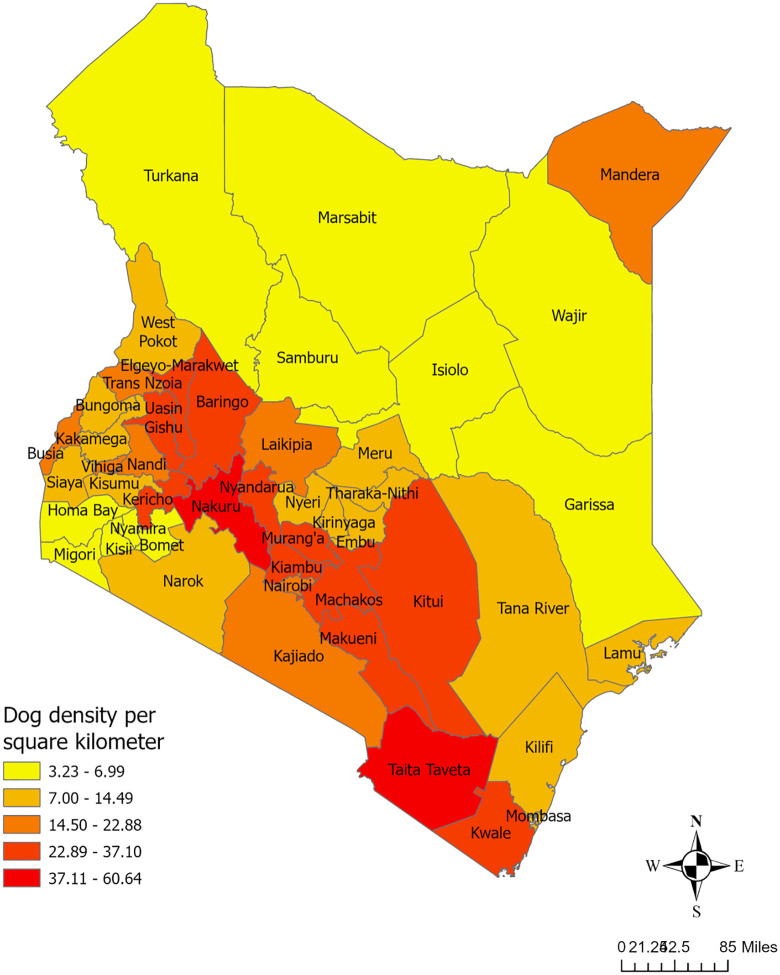
Predicted spatial distribution of free-roaming dog density (dogs/km²) in Kenya. Predictions are based on the final co-kriging model incorporating human density as a covariate.

**Fig 3:**
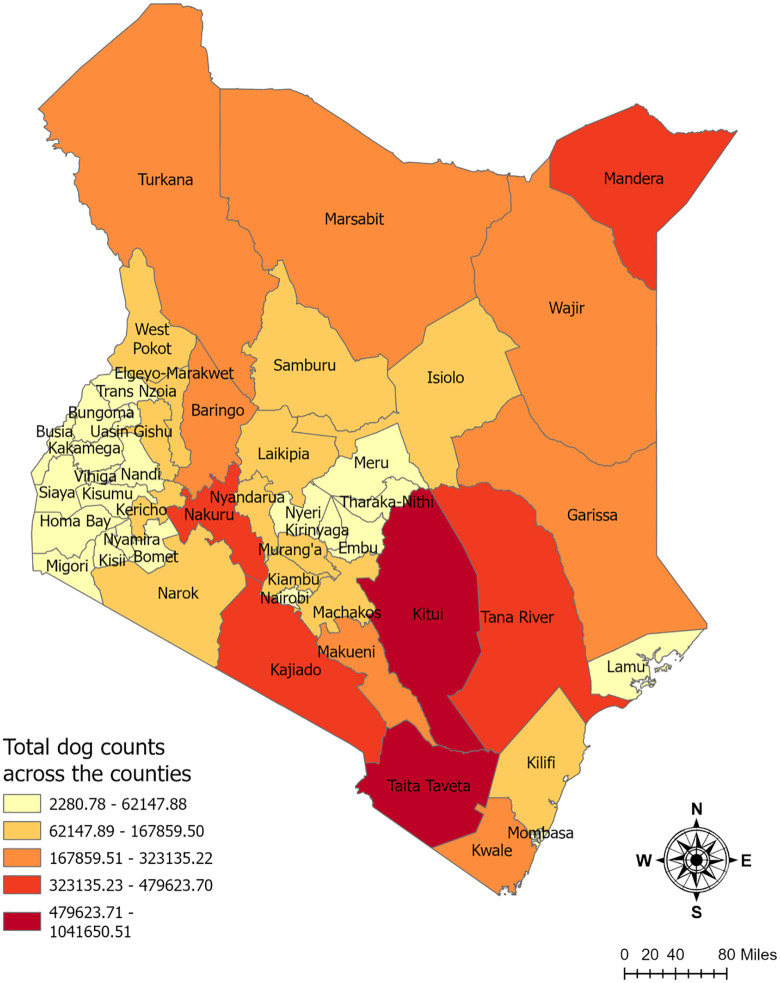
Total estimated free-roaming dog population counts per county. These values were calculated by multiplying the predicted density from Figure 2 by the area of each county.

The dog density per square kilometer (Fig 2) and total number of free-roaming dogs in Kenya (Fig 3) were derived from the summary statistics of the best-fitted model. Our analysis estimated a total of 7.46 million dogs across all 47 counties, with a median density of 12.13 dogs per km² in Kenya. The counties with relatively higher dog densities ranging from 31 to 60 dogs per square kilometer were Taita Taveta, Nakuru, Makueni, Kwale, and Kiambu. In contrast, lower dog densities, ranging from 3 to 7 dogs per square kilometer, were recorded in Marsabit, Migori, Kisii, Nyamira, Garissa, Bomet, Wajir, Samburu, Isiolo, Turkana and Homa Bay. Among all counties, Taita Taveta had the highest total dog population, while Marsabit recorded the lowest number of dogs (S1 Table).

## Discussion

The present study aimed to predict the number of free-roaming dogs in Kenya using data from 34 counties. The best-fitted model identified human density as the only predictor of dog density, highlighting the close relationship between human presence and free-roaming dog populations. Our results indicate significant variation in dog densities across Kenya, with the highest densities observed in urban and peri-urban counties such as Taita Taveta, Nakuru, Makueni, Kwale, and Kiambu. In contrast, the lowest densities were recorded in sparsely populated, arid, and pastoralist regions, including Marsabit, Turkana, Garissa, Isiolo, Wajir, and Samburu. This finding suggests that dog populations are more concentrated in areas with higher human density, where food availability and human-dog interactions are more prevalent. At the same time, pastoralist and arid regions support lower dog densities, likely due to resource limitations and nomadic lifestyles. It is essential to acknowledge that our predictions provide an estimate rather than exact numbers, and actual dog densities in some counties may differ from our projections. This variation may partly stem from the subjective nature of the original data collection, which could introduce random errors into the estimates. While these errors are difficult to quantify, we believe they are not systematic in nature. Instead, they are likely to be random and localized, resulting from factors such as community-specific practices, environmental variability, or unaccounted food sources. To address potential inconsistencies, our modeling approach incorporates spatial smoothing by weighting observations from nearby locations. This technique helps balance out potential over- and under-estimates associated with random error, enhancing the reliability of the overall predictions.

The current study’s estimated total dog population in Kenya (7.46 million) aligns closely with the previous approximations in Kenya’s strategic plan for the elimination of human rabies (5–6 million) estimated in 2014 [33]. The former estimate in the country’s strategic plan lacks clear documentation on how it was calculated, making it challenging to validate its accuracy. In contrast, 7.46 million approximations in the present study are based on a spatial analysis approach (kriging/co-kriging) that integrates geographic and environmental factors, likely leading to a more sophisticated and evidence-based estimate. According to the United Nations, Kenya’s population is growing by approximately one million annually [34]. If the human-to-dog ratio remains consistent, it is reasonable to expect a corresponding increase in the dog population over time. This result suggests that the estimated 5–6 million dogs in 2014 may align well with the estimated 7.46 million dogs in this study.

According to the model, Taita Taveta county exhibits the highest dog density. Located in the southwestern coastal region of Kenya, 62% of the county is covered by two national parks, while humans inhabit the remainder. Taita Taveta shares its borders with six other counties in Kenya and with the Republic of Tanzania to the southwest [35]. The human-to-dog ratio in this Taita Taveta county was approximately 0.33, indicating that there was one human for every three dogs. This might be due to a single household owning multiple dogs, which may lead to a high dog-to-human ratio. In countryside areas, keeping multiple dogs is a part of the culture, unlike in urban cities, where open space is available [36]. In Taita Taveta county, livestock rearing is one of the primary sources of income activities [37]. Alternatively, the elevated prediction of dog density in this county may be explained, at least in part, as an overestimation resulting from an edge effect in the methods used to analyze the data. In such cases, the model may artificially inflate estimates or distort predictions due to the altered environmental conditions near the county boundaries of the assessed territory [38]. A key benefit of this model is the ability to compare its predictions against the localized, ground-based studies mentioned previously. For Machakos county, an earlier study reported a wide density range of 6–110 dogs per square kilometer [19]. Our model’s estimate for Machakos was 25.5 dogs/km² (S1 Table), a figure that falls comfortably within this previously established range. In western Kenya, a prior study estimated 54 dogs per square kilometer [20]. Our model, in contrast, predicted lower densities for counties in this region, such as Kisumu (12.2 dogs/km²) and Kakamega (11.8 dogs/km²) (S1 Table). This discrepancy is likely attributable to the significant differences in methodology; the prior study was an intensive localized demographic surveillance survey, whereas our model is a geostatistical interpolation of expert-reported estimates. Finally, while a prior household survey in Laikipia estimated a total count of 34,275 dogs [21], our model provides a county-wide density estimate of 17.5 dogs/km² (S1 Table), offering a new, spatially explicit perspective. These comparisons suggest our model aligns with some local estimates while also highlighting how methodological differences can impact population estimations.

Some of Kenya’s central and southeastern counties, including Nakuru, Makueni, Kiambu, Kericho, and Nyandarua, were identified as having higher dog capacities. This pattern can be attributed to a combination of socioeconomic and environmental factors that influence the distribution and survival of free-roaming dogs. Among these counties, Kiambu, Nakuru, and Makueni have significant human populations, with a mix of urban, peri-urban, and rural settlements. These densely populated areas might provide higher food availability for free-roaming dogs, primarily through restaurant leftovers, public waste, and garbage dumps. Studies have shown that free-roaming dog populations tend to be more abundant, where food waste is more accessible [39,40]. Lower-income neighborhoods and slum areas often have more open spaces, fewer restrictions on animal movement, and a high tolerance for free-roaming dogs, leading to a higher capacity for sustaining dog populations [41]. On the other hand, Kericho and Nyandarua are predominantly agricultural counties, where farming and livestock rearing play a central role in livelihoods [42,43]. In such agriculture-based areas, dogs are commonly used for security, livestock guarding, and protection of farmlands against intruders or wild animals. Similar patterns of higher dog densities in agricultural communities have been observed in pastoralist regions such as Tanzania [44] and Turkana, Kenya [45], where dogs serve as herding aids, protectors of livestock, and household guardians. Additionally, climatic and environmental factors may contribute to these counties’ higher dog capacities. Kericho and Nyandarua have favorable climatic conditions with moderate rainfall, vegetation cover, and food availability, which can sustain larger free-roaming dog populations [43].

Several urbanized counties in Kenya, including Nairobi, Mombasa, Kisumu, and Kajiado, exhibited moderate to low dog densities compared to more rural or agricultural regions. The distribution of dog populations in urban settings is influenced by structured pet ownership, municipal regulations, waste management systems, and socioeconomic factors. Urban municipalities actively control stray dog populations through vaccination, sterilization, and culling programs to prevent the spread of diseases [46]. This finding aligns with the study from Uganda, which states that areas with high population density and high poverty tend to have a higher number of dogs [47]. A similar finding is recorded from a study conducted in Chili, stating that the higher the human density, the higher the turnover of the free-roaming domestic dogs [48].

Dog densities have been recorded at their lowest levels in several counties across north-eastern Kenya, including Marsabit, Turkana, Samburu, Wajir, Isiolo, and Garissa, as well as in some western counties such as Homa Bay, Migori, Kisii, Nyamira, and Bomet. One major factor contributing to these low densities is the pastoral lifestyle that is dominant in many arid and semi-arid regions, particularly in counties like Turkana, Isiolo, and Garissa. Pastoralist communities in these areas traditionally migrate seasonally for water and pasture, moving alongside their livestock and pets [49]. Due to the dynamic nature of the human population, it might be challenging to predict the actual number of dogs in this community. Another key factor influencing dog densities is religious and cultural preferences. Counties such as Garissa and Wajir have a predominantly Muslim population, and in Islamic tradition, dog ownership is generally discouraged [50]. Since most dogs in Kenya are owned but free-roaming [20], the religious restrictions on dog ownership in these counties could contribute to their lower overall densities. Furthermore, in highly urbanized or agriculturally focused regions like Kisii, Nyamira, and Bomet, the availability of land for roaming dogs is limited, and the cultural significance of dog ownership is relatively lower compared to other regions.

## Limitations of the study

The findings of this study must be interpreted within the context of its significant limitations, which are essential for guiding future research. The primary constraint is the data source: expert-reported estimates, which carry a high potential for response bias and were not empirically validated with ground-truthing (e.g., mark-resight surveys) due to resource constraints. This study should therefore be considered a foundational, cost-effective framework, not an empirical census. This data sparsity also means model uncertainty is highest in the 13 unsampled counties where the model had to extrapolate, a limitation not visually quantified as a kriging standard error map is absent. Other acknowledged methodological constraints include a likely “edge effect” causing overestimation in Taita Taveta, minor measurement bias from village area approximations, and the absence of a full sensitivity analysis or alternative cross-validation metrics (e.g., MSE). Despite these acknowledged constraints, this study provides the first national-level geostatistical baseline for Kenya. It identifies high-priority hotspots and high-uncertainty regions, thereby creating the essential spatial framework to guide and justify future, resource-intensive validation, surveillance, and public health interventions. Addressing these challenges in future research will further improve model accuracy and applicability.

## Conclusions

The present study provides a comprehensive spatial analysis of free-roaming dog populations across Kenya, addressing the longstanding gap in standardized population assessment methods. By incorporating environmental and demographic predictors, we refined spatial predictions, resulting in a national free-roaming dog estimate with an average density of dogs per square kilometer. The findings reveal regional variations in dog densities across Kenya. Counties with moderate-to-high human densities exhibited higher dog capacities, likely due to increased food availability, mixed urban-peri-urban settings, and agricultural activities that support dog ownership. In contrast, dog densities were lowest in sparsely populated, arid, and pastoralist counties, where harsh climatic conditions, nomadic lifestyles, and cultural factors limit dog populations. Additionally, some urban counties exhibited moderate-to-low dog densities, potentially due to structured pet ownership, municipal regulations, and effective stray management programs. Overall, these findings establish a spatial framework for estimating free-roaming dog populations in Kenya, which can inform targeted rabies vaccination campaigns, resource allocation, and strategic public health planning. Future studies should integrate longitudinal data and additional ecological factors to enhance predictive accuracy further and support sustainable dog population management. Continued refinement of predictive models, and ground-truth validation, and adaptive management strategies will be essential for sustaining rabies elimination efforts and improving dog population management across Kenya.

## Supporting information

S1 Table Predicted density of dogs per km^2^ (lowest to highest) in 47 counties in Kenya. (DOCX)

S2 File Inclusivity-in-global-research-questionnaire. (DOCX)
