# Supplementary material for: Spatial prediction of dog population distribution in Kenya
Source: PLoS One. 2026 Apr 13;21(4):e0343347. doi: 10.1371/journal.pone.0343347 (PMC13075693; doi:10.1371/journal.pone.0343347)
Supplement: S1 Table — (DOCX) [file pone.0343347.s001.docx]

**S1 table:** Predicted density of dogs per km^2^ in 47 counties in Kenya, sorted from lowest (Marsabit) to highest (Taita Taveta).

| **County** | **Density of dogs** |
| --- | --- |
| Marsabit | 3.232146 |
| Migori | 3.449446 |
| Kisii | 3.525154 |
| Nyamira | 3.626622 |
| Garissa | 4.468984 |
| Bomet | 4.896435 |
| Wajir | 4.994374 |
| Samburu | 5.175637 |
| Isiolo | 5.348895 |
| Turkana | 5.391844 |
| Homa Bay | 6.989072 |
| Tharaka-Nithi | 8.241109 |
| Meru | 8.339488 |
| Narok | 8.762831 |
| Mombasa | 9.540445 |
| Kirinyaga | 10.18461 |
| Lamu | 10.21465 |
| Nyeri | 10.90325 |
| Embu | 11.08107 |
| Kilifi | 11.12947 |
| Kakamega | 11.75128 |
| Siaya | 11.84782 |
| Tana River | 12.02671 |
| Kisumu | 12.18701 |
| West Pokot | 13.4379 |
| Bungoma | 14.49347 |
| Busia | 15.69662 |
| Vihiga | 16.02044 |
| Trans Nzoia | 17.12211 |
| Laikipia | 17.53187 |
| Mandera | 18.78171 |
| Nandi | 20.31394 |
| Kajiado | 21.90231 |
| Nairobi | 22.87956 |
| Uasin Gishu | 24.36392 |
| Kitui | 24.62489 |
| Machakos | 25.50593 |
| Elgeyo-Marakwet | 26.70935 |
| Murang'a | 28.29984 |
| Baringo | 29.57692 |
| Nyandarua | 29.79263 |
| Kericho | 29.88228 |
| Kiambu | 31.10806 |
| Kwale | 34.05886 |
| Makueni | 37.0996 |
| Nakuru | 50.1318 |
| Taita Taveta | 60.64291 |
